# Treatment of Odontogenic Myxoma: A Multidisciplinary Approach—6-Year Follow-Up Case

**DOI:** 10.1155/2014/795808

**Published:** 2014-12-14

**Authors:** João Gustavo Oliveira de Souza, Jonathas Daniel Paggi Claus, Felipe Damerau Ouriques, Luiz Fernando Gil, José Nazareno Gil, Antonio Carlos Cardoso, Marco Aurélio Bianchini

**Affiliations:** ^1^Department of Implant Dentistry, School of Dentistry, Federal University of Santa Catarina, Rua Frei Evaristo 64, apto 703, Centro, 88015-410 Florianópolis, SC, Brazil; ^2^Residence of Oral and Maxillofacial Surgery Program, HU/UFSC, Rua Frei Evaristo 64, apto 703, Centro, 88015-410 Florianópolis, SC, Brazil; ^3^Department of Oral and Maxillofacial Surgery, School of Dentistry, Federal University of Santa Catarina, Rua Frei Evaristo 64, apto 703, Centro, 88015-410 Florianópolis, SC, Brazil

## Abstract

The most aggressive diseases that affect the oral environment are considered tumors of the jaw. The surgical treatment is preferably done by surgical resection of the lesion, resulting in a great loss of tissue and esthetics. Multidisciplinary planning is required for the rehabilitation of these cases. Autogenous grafting techniques or vascularized flaps allow ridge reconstruction for implant placement, restoring function, and esthetics. This paper reports a 6-year follow-up case of an odontogenic myxoma treated with wide resection and mandibular bone reconstruction for posterior rehabilitation with dental implants.

## 1. Introduction

Odontogenic myxoma is a rare benign tumor unencapsulated aggressive and located character, originating from mesenchymal cells. Although both jaws can be affected, this tumor is slightly more common in the mandible [1–3]. If the treatment is neglected, it can result in complications with an increased possibility of morphological and functional damage. Treatment and therapeutic modalities vary from curettage to large block resections and subsequently bone grafts for reestablishment of hard tissue.

The skull, iliac crest, rib, and tibia are the most commonly used [4] for bone reconstruction due to the need of large amounts, particulate or bone blocks. The rehabilitation of these cases is complicated by the necessity for bone reconstruction and soft components to allow correct support for the facial tissue and multidisciplinary professionals.

There is no consensus in the literature between autogenous-free bone grafts and vascularized grafts. Vascularized grafts show less rate of resorption, presenting higher proportional success for implants. On the other hand, it increases the time of the surgery and hospitalization, as well as the cost of treatment and the postoperative morbidity [4, 5].

Implant-supported prosthesis (ISP) seems to be the best alternative for these cases due to the unfavorable features of the grafted area, extreme mobile mucosa and lack of vestibule. Moreover, compared to removable prostheses, ISPs offer better masticatory capacity, patient comfort, and speech improvement, among others [6].

The present paper, through a 6-year follow-up case, will focus upon the management of an odontogenic myxoma treated with wide resection and mandibular bone reconstruction for posterior rehabilitation with dental implants.

## 2. Case Report

**Figure 1:**
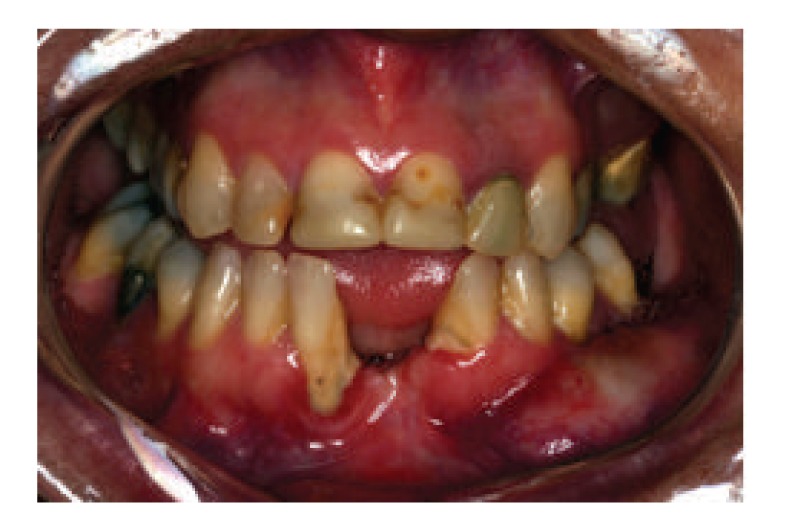
Initial frontal view.

**Figure 2:**
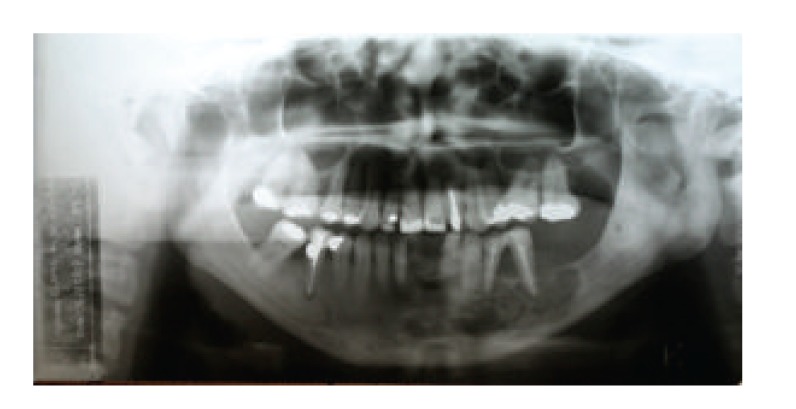
X-ray showing the lesion limits.

A 36-year-old female patient was attended to with chief concern of swelling in her lower jaw (Figure 1). After completion of clinical and radiographic examination, a biopsy was performed, confirming the diagnosis of odontogenic myxoma (Figure 2). After a computed tomography (CT), the lesion limits were determined and a 3D prototype model was elaborated for planning the mandibulectomy and selecting and shaping the reconstruction plate.

**Figure 3:**
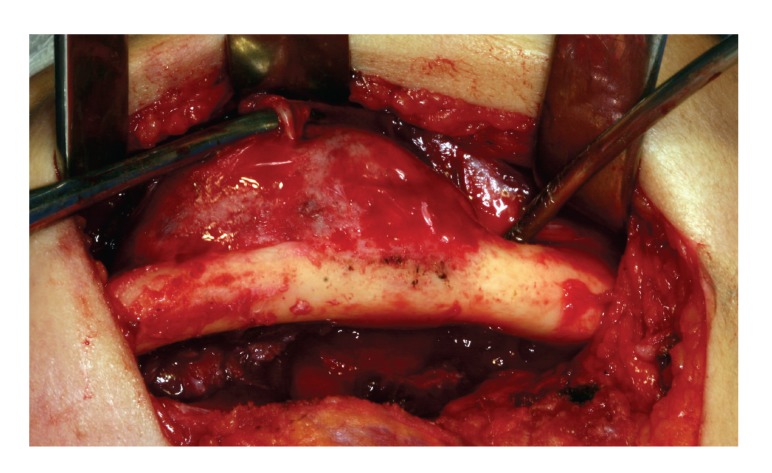
Transoperative view of the tumor.

**Figure 4:**
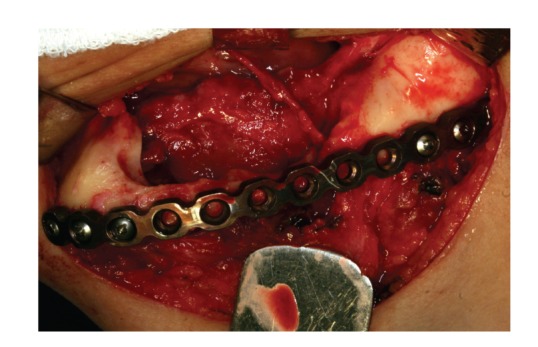
Transoperative view after tumor resection.

**Figure 5:**
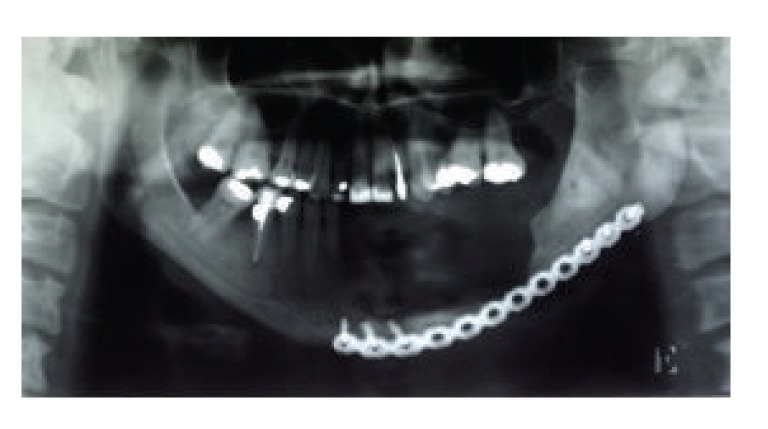
Postoperative X-ray showing surgical margins and the continuity of the base of the mandible.

The patient underwent segmental mandibulectomy, under general anesthesia, from the left ramus to the symphysis, by a surgical resection including a safety margin of 1.5 cm (Figures 3 and 4). The patient was followed up with clinical and radiographic control (Figure 5).

Two months later, the patient underwent reconstruction with bone graft, from the right lateral iliac crest, only via extra oral submandibular access to eliminate the intraoral contamination (Figure 5). Intermaxillary fixation was applied for one month in order to reduce mobility to increase the predictability of the grafting.

**Figure 6:**
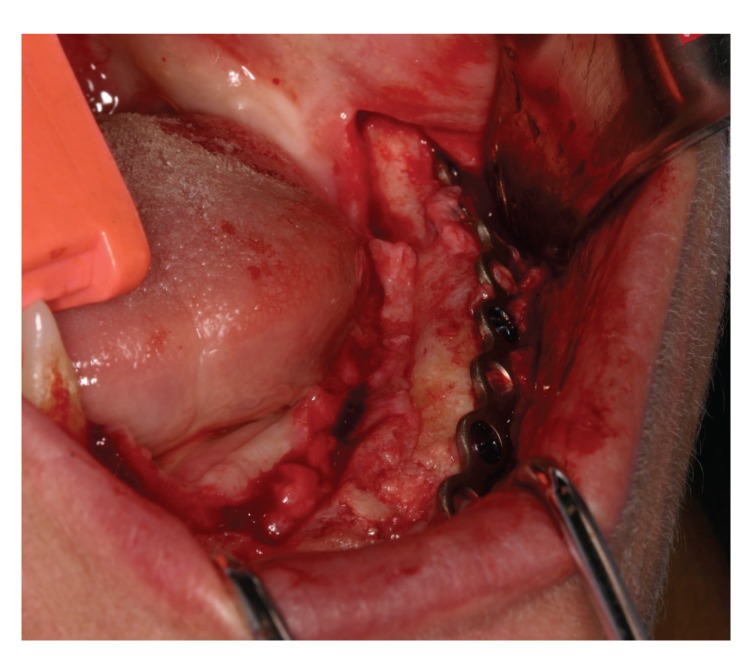
Reconstruction to increase the bone height to allow implant placement.

**Figure 7:**
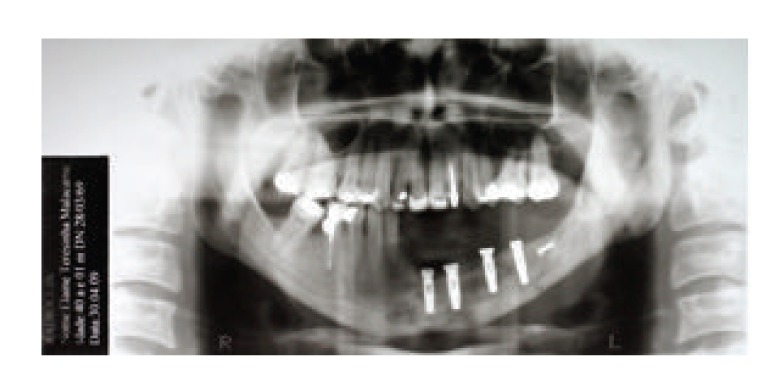
Panoramic radiographic showing implants placed in position.

**Figure 8:**
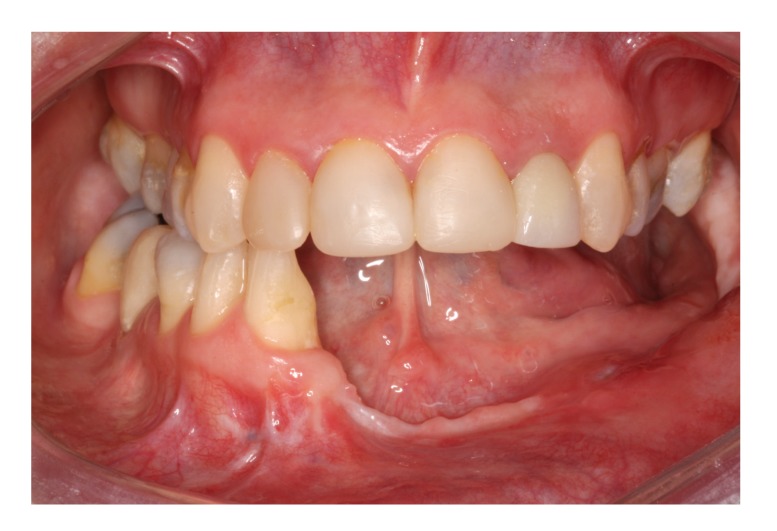
Front view before second surgery stage.

After six months, a CT scan was requested for implant planning (Figure 6). Four implants of 3.75 × 11 mm diameter (Neodent Company, Brazil) were placed in the grafted area under local anesthesia (Figure 7). The second surgery stage was performed six months later and restoration was started (Figure 8). Any soft tissue surgery was performed to increase the width or keratinized mucosa (KM) [7].

**Figure 9:**
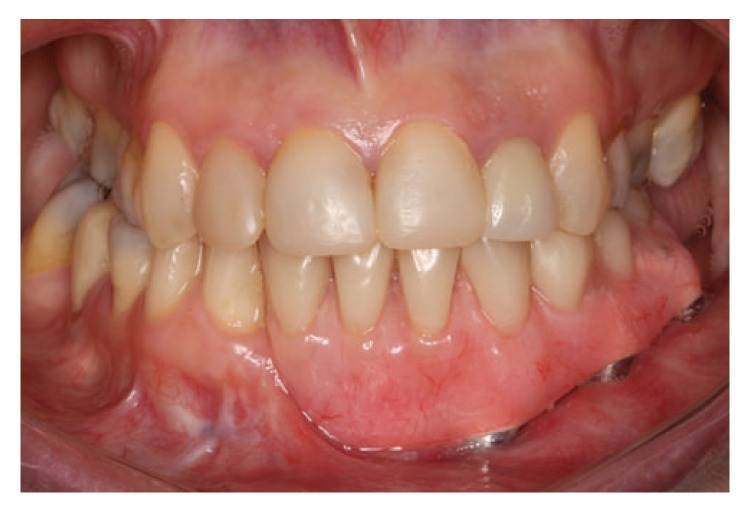
Prosthesis installed 1 year after tumor resection.

Implant abutments similar to multiunit were selected. The impression of the abutments was taken with a custom tray and polyether impression material (Impregum Soft, 3M ESPE, Germany). In the next step, the patient was handled in centric relation to the contact to the antagonist arch, in the same vertical dimension of occlusion of the patient. After confirming the settlement of the metal structure and evidence of the teeth, the prosthesis was finally cured and installed (Figure 9).

**Figure 10:**
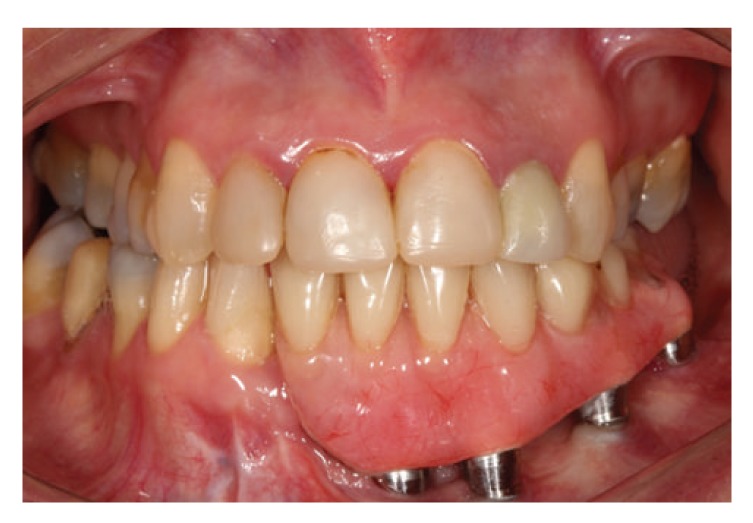
Six-year follow-up after tumor resection, clinical view.

**Figure 11:**
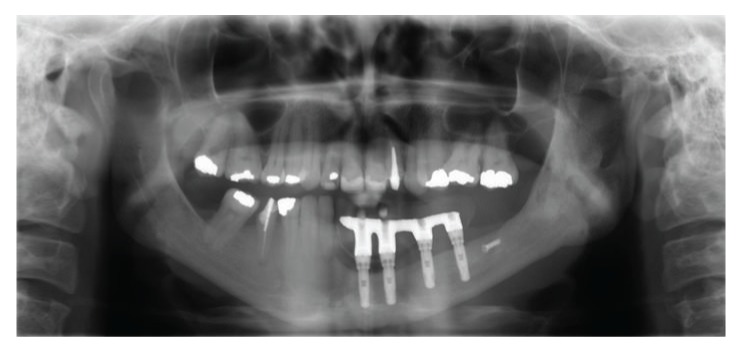
Panoramic radiographic 6 years after tumor resection.

The patient was clinically and radiographically followed up over 6 years without evidence of recurrence of the lesion with stability of the peri-implant bone tissue; all implants showed probing depth less than 3 mm, absence of visible biofilm, and no bleeding probing (Figures 10 and 11).

## 3. Discussion

Resection of malignant or benign tumors of the jaw causes emotional, functional, and aesthetic complications if not diagnosed and treated properly. Rehabilitation consists of different options for bone grafting, type and origin of the graft, and the use or not of fixed prostheses and implants.

Use of vascularized grafts, in no larger than 6 cm bone defects, is not advocated by many authors due to high failure rates. However, a recent study obtained favorable results with the use of vascularized grafts not for large bone defects [8]. The disadvantage of using nonvascularized iliac crest bone grafts is its high rate of resorption [5]. However, a greater interaction creates a correlation between the placement of graft resorption and implant. There is a decrease in bone resorption due to the functional matrix theory [7, 8].

The decision not to use vascular grafts is due to the excellent results and the authors of this work, with free grafts, performed the following with some basics: (1) tumor resection and bone graft at different surgical times (need for an intraoral approach for resection and contamination), graft after two months with only extraoral submandibular approach; (2) iliac crest bone graft (quality and quantity); (3) intermaxillary fixation to reduce local mobility; (4) avoiding dentures on the grafted areas. At the time of the first surgery, the authors did not have this protocol and this case has contributed to such information in accordance with the extensive resorption of the first graft. A 6-year follow-up has shown no recurrence, stable prosthesis, and health of peri-implant soft tissues. Although some expected graft resorption took place, there is no clinical or radiographic sign of implant failure. These results are similar to the case report presented by Yoshimura et al. (2013) [9].

Second reconstruction was performed aiming at fixed prosthodontics supported by long implants. This rehabilitation has to accomplish functional and esthetical outcomes. Despite the amount of artificial gengiva the result is biomechanically and biologically stable and patient is satisfied.

Several studies show that Perio-implant health can be maintained even in places without KM provide adequate control plate [10–12]. It was also observed that only the presence of a wide range of KM cannot result in an improvement of the peri-implant parameters when compared to sites with little or no amount of KM.

Oral rehabilitation of traumatized patients, infection, and/or tumor is a challenge that requires multidisciplinary treatment. The success rate of implants in reconstructed patients, regardless of the type of prosthesis used, is similar to the installed implants in healthy patients. It also happens when comparing the success of implants supporting overdentures or fixed prostheses [13].

Moreover patients who underwent segmental reconstruction should be informed about increased risk of marginal bone loss before implant placement. Professionals should perform a detailed examination during maintenance consultations and also maintain a track of the clinical parameters to allow proper control of additional bone loss progression.

This paper reports a case, which showed the possibility of rehabilitating patient's function and esthetics, using bone grafts, implants, and fixed prosthesis.
